# miR-6869-5p Transported by Plasma Extracellular Vesicles Mediates Renal Tubule Injury and Renin-Angiotensin System Activation in Obesity

**DOI:** 10.3389/fmed.2021.725598

**Published:** 2021-09-08

**Authors:** Huan-Huan Liu, Xia-Qing Li, Jin-Feng Liu, Shuang Cui, Han Liu, Bo Hu, Si-Bo Huang, Li Wang, Wah Yang, Cun-Chuan Wang, Yu Meng

**Affiliations:** ^1^Department of Nephrology, The First Affiliated Hospital of Jinan University, Guangzhou, China; ^2^Nephrology Department, Southern Medical University Affiliated Longhua People's Hospital, Shenzhen, China; ^3^Department of Metabolic and Bariatric Surgery, The First Affiliated Hospital of Jinan University, Guangzhou, China; ^4^Jinan University Institute of Obesity and Metabolic Disorders, Guangzhou, China; ^5^Central Laboratory, The Fifth Affiliated Hospital of Jinan University, Heyuan, China; ^6^Jinan University Institute of Nephrology, Guangzhou, China

**Keywords:** miR-6869-5p, extracellular vesicles, obesity, renal tubule injury, renin- angiotensin system

## Abstract

Obesity increases the risk of other diseases, including kidney disease. Local renal tubular renin-angiotensin system (RAS) activation may play a role in obesity-associated kidney disease. Extracellular vehicles (EVs) transmit necessary information in obesity and cause remote organ damage, but the mechanism is unclear. The aim of the study was to investigate whether the plasma EVs cargo miR-6869-5p causes RAS activation and renal tubular damage. We isolated plasma EVs from obese and lean subjects and analyzed differentially-expressed miRNAs using RNA-seq. Then, EVs were co-cultured with human proximal renal tubular epithelial cells (PTECs) *in vitro*. Immunohistochemical pathology was used to assess the degree of RAS activation and tubule injury *in vivo*. The tubule damage-associated protein and RAS activation components were detected by Western blot. Obesity led to renal tubule injury and RAS activation in humans and mice. Obese-EVs induce RAS activation and renal tubular injury in PTECs. Importantly, miR-6869-5p-treated PTECs caused RAS activation and renal tubular injury, similar to Obese-EVs. Inhibiting miR-6869-5p decreased RAS activation and renal tubular damage. Our findings indicate that plasma Obese-EVs induce renal tubule injury and RAS activation via miR-6869-5p transport. Thus, miR-6869-5p in plasma Obese-EVs could be a therapeutic target for local RAS activation in obesity-associated kidney disease.

## Introduction

Obesity is a worldwide disease that increases the risk of other diseases and health problems (1). Abnormal fat deposition causes various metabolic abnormalities, including dyslipidemia, insulin resistance, atherosclerosis, and hypertension (2). Renal lipid accumulation and lipotoxicity contribute to kidney cell injury and death, and eventually kidney disease (3). Previous reports indicate that renal tubular changes, including hypertrophy (4) and vacuolar degeneration (5), can be seen in subjects with obesity-related kidney disease. Other studies have reported that extracellular vesicle (EV) secretion in obesity plays an essential role in kidney diseases (6, 7). We previously described the involvement of EVs in the pathophysiology of glomerular diseases (8). However, the role of EVs in obesity-related kidney disease, especially for renal tubule injury, is poorly understood.

Dysregulation of the renin-angiotensin system (RAS) has a critical role in the pathogenesis of kidney injury in obesity (9). The classical RAS cascade starts with prorenin production, an aspartate protease secreted by renal juxtaglomerular cells in response to decreased circulating blood volume. Kalupahana et al. reported RAS overactivation in obese patients or obese animal models. However, angiotensin-converting enzyme inhibitors (ACEI) or angiotensin receptor blockers (ARB) decreased adipocyte size. Consistently, they also found discrepancies in RAS component expression in these models (10, 11). Excessive RAS activation leads to increased angiotensin II (AngII), which leads to increased kidney oxidative stress damage, inflammation, and fibrosis. AngII promotes increased expression of transforming growth factor-β1 (TGF-β1) and type I collagen in the kidney, leading to severe renal damage, glomerular sclerosis, and interstitial fibrosis (12). RAS activity affects the expression of EV proteins derived from kidneys, which regulate RAS (13).

EVs are membrane-bound vesicles secreted by all cells, and they contain protein, miRNA, mRNA, and other substances. MicroRNA can be transferred between cells via EVs, thus exerting post-transcriptional regulatory functions (14). The EV contents significantly increase in chronic kidney disease and uncomplicated obese patients (15, 16). MicroRNA analysis of EVs extracted from mice with kidney disease revealed a significantly differential expression. Consistently, almost all the detected miRNAs are related to kidney damage (17). Thus, miRNA expression may be regulated indirectly by RAS and may significantly affect kidney injury, leading to higher renal fibrosis and proteinuria (18).

We investigated the role of the EVs cargo miR-6869-5p in obesity with kidney injury *in vitro* and *in vivo*. We hypothesized that plasma EVs derived from obese patients might participate in RAS activation and kidney injury. Herein, we isolated plasma EVs in obese and lean participants and incubated renal proximal tubular epithelial cells (PTECs) with the isolated EVs to detect RAS activation and biomarkers of renal tubular injury. RNA sequencing on Obese- and Lean-EVs showed a significant upregulation of miR-6869-5p in Obese-EVs vs. Lean-EVs. miR-6869-5p in Obese-EVs can be quickly delivered into PTECs, thus inducing RAS activation and renal tubular damage. This effect can be significantly reduced after the miR-6869-5p was silenced. Therefore, miR-6869-5p could mediate renal tubule injury and RAS activation in obesity.

## Materials and Methods

### Patients

The study was approved by the Ethics Committee of the First Affiliated Hospital of Jinan University (Permission No. 2018-041). Written informed consent was obtained from all participants. A total of 50 participants, including 25 healthy volunteers and 25 obese patients, were enrolled at the First Affiliated Hospital of Jinan University. We recruited lean participants with no metabolic syndrome components (National Cholesterol Education Program, Adult Treatment Panel III criteria). In the obese groups, the inclusion criteria were as follows: (1) The patients undergoing bariatric surgery with a body mass index (BMI) ≥ 30 kg/m^2^, according to the WHO criteria; (2) Age >18, but <80 years at the diagnosis of obesity; (3) Availability of complete clinical data and follow-up status. The exclusion criteria were: patients with a BMI > 55 kg/m^2^, cancer, pregnancy, severe infection, history of alcoholism or drug abuse, severe organic diseases, and incomplete clinical data. Data on clinical variables, including sex, age, family and personal history, and selected laboratory results were gathered.

### Cell Culture

The human renal PTEC line, human kidney-2 (HK2) cells, were obtained from the Chinese Academy of Sciences Cell Bank (Shanghai, China). HK2 cells were cultured in Dulbecco's modified Eagle's medium (DMEM)/Hams F12 (1:1) (01-172-1ACS, Biological Industries) supplemented with 10% fetal bovine serum (FBS) (Gibco, Grand Island, NY, USA) and 1% penicillin-streptomycin (Gibco, USA), and cultured under standard conditions (37°C and 5% CO2). Cells were counted using a hemocytometer. The medium was replaced daily.

### Animals

Male C57BL/6J mice (20–25 g, 4 weeks old) were obtained from SPF Biotechnology Co., Ltd (Beijing, China). Animals (*n* = 5 per group) were randomly divided into control and high-fat diet (HFD) groups. The experimental protocols were approved by the Institutional Animal Care and Use Committee of Jinan University (No. 32219008). The mice were maintained in a controlled environment (23 ± 2°C, 12-h light/dark cycle) with access to food and water *ad libitum*. Mice received two different dietary regimens: (1) a standard chow diet and drinking water, and (2) an HFD diet (20.6% carbohydrate, 60.0% lipid, and 19.4% protein, #TP23300, TROPHIC Animal Feed High-tech Co., Ltd. Nantong, China) and 10% fructose (Sigma-Aldrich) in the drinking water (19). The mice were weighed weekly from the beginning of the study. After 18 weeks, all the mice were fasted for 12 h and sacrificed. Blood samples were taken from the inferior vena cava, and the kidneys were collected.

### Plasma EV Isolation and Characterization

**Figure 1:**
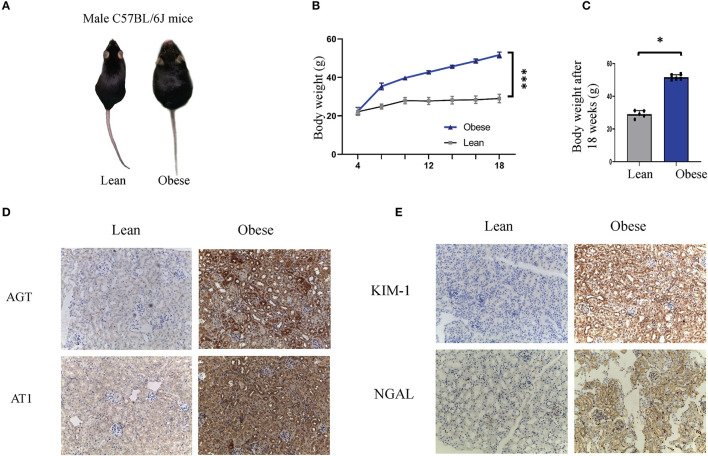
Obesity leads to renal tubule injury and RAS activation in humans and mice. **(A–C)** Representative images **(A)**, body weight curves **(B)**, and bodyweight levels after 18 weeks **(C)**, of obese and lean mice. **(D,E)** Representative immunohistochemical staining of AGT, AT1, KIM1, and NGAL in kidney sections of lean and obese mice (Original magnification 200×). AGT, angiotensinogen; AT1, angiotensin 1; KIM-1, kidney injury molecule-1; NGAL, neutrophil gelatinase-associated lipocalin. *P < 0.001; ***P < 0.05.

Plasma EV isolation and identification followed the MISEV 2018 guidelines (20, 21), as described in previous studies (22–25). Figure 1 shows the schema for isolation of plasma EVs by ultracentrifugation (UC) and size-exclusion chromatography (SEC). Briefly, 50 mL human peripheral blood (10 mL blood from five participants per group were pooled) was obtained from the participants in obese and lean groups. All blood samples were processed within 1 h of collection. For plasma preparation, the blood samples were centrifuged at 1,300 × g for 10 min. Then, the plasma underwent successive centrifugations (4,000 × g for 15 min, and 15,000 × g for 60 min). The supernatant was collected and then ultracentrifuged for 60 min at 100,000 × g on the same day. The EV-containing pellet was washed twice with PBS and resuspended in 15 mL PBS. The final suspension was filtered through a 0.22 um-pore filter (Millipore, Billerica, USA), followed by SEC using 1.5 ×12 cm columns (Bio-Rad, Hercules, CA, USA; Econo-Pac columns) packed with Sepharose 2B (Sigma-Aldrich, St. Louis, MO, USA). The eluate was collected, and the presence of EV was verified. The EVs were immediately used for experiments or stored at −80°C. EVs were identified based on CD9, CD63, and CD81 expression and analyzed by Western blotting, as described previously (25, 26). The EV morphology was evaluated using transmission electron microscopy (TEM) (JEM-1400, Japan), as previously described (26). The particle size distribution and EV concentrations were measured by nanoparticle tracking analysis (NTA) (27–29).

### *In vitro* EV Treatment

For *in vitro* assays, PTECs (80–90% confluent) were seeded in 6-well cell culture plates and were co-cultured with PBS (vehicle), 20 μg Lean-EVs, or 20 μg Obese-EVs for 48 h (19). Then, the cells were collected by trypsin (0.25%, Invitrogen, USA) digestion, as previously described (30).

### MicroRNA Sequencing and Data Analysis

MicroRNA sequencing was performed as previously described (31, 32). Briefly, total RNA from EVs was extracted. RNA-seq libraries were prepared from the extracted RNA using QIAseq Stranded Total RNA Kits and sequenced with an Illumina NGS system (MiSeq Personal Sequencer, NextSequence500, HiSeq-1000, HiSeq-1500, HiSeq-2000, HiSeq-2500, and GaIIx). The RNA-seq data were analyzed using the CLC (Biomedical) Genomics Workbench. Starting with unaligned FASTQs, the workflow generates aligned BAMs and then both raw and normalized miRNA expression counts. Differential expression analysis was performed using EdgeR 2.6.2 software.

### *In vitro* miR-6869-5p Transfection

The miR-6869-5p mimic (20 nM, 5′-GUGAGUAGUGGCGCGCGGCGGC-3′) and miR-6869-5p inhibitor (20 nM, 5′-GCCGCCGCGCGCCACUACUCAC-3′) were synthesized by Guangzhou Ribobio Co., Ltd. (Guangzhou, China). PTECs grown on 6-well plates to 40–50% confluence were transfected using Lipofectamine® 3000 Reagent (Cat#L3000015, Invitrogen, Carlsbad, CA, USA) in FBS-free Opti-MEM (Gibco, USA), according to the manufacturer's instructions. The medium was replaced after 24 h. The transfected cells were harvested 48 h later for further experiments. The transfection efficiency was tested by qRT-PCR.

### qRT-PCR Assays

Total RNA from plasma EVs and PTECs (miR-6869-5p-transfected and EV-treated cells) was extracted using TRIzol reagent (Thermo Fisher Scientific, Inc.) (33). Then, the RNA was reverse transcribed using RevertAid First Strand cDNA Synthesis Kits (K1622, Thermo Scientific). qRT-PCR was carried out using SYBR Green PCR Master Mix (Applied Biosystems, USA) and a BioRad CFX-96 real-time PCR system (BioRad, USA). The primers are listed in Table 1. The miRNA and mRNA levels were normalized to U6 and GAPDH levels, respectively. Relative RNA expression was calculated using the 2^−ΔΔCT^ method.

**Table 1:** The list of primers sequences.

**RT-PCR primers**	**Forward Primer (5′-3′)**	**Reverse Primer (5′-3′)**
AGT	CAGGCTGTGACAGGATGGAA	GCTGTTGTCCACCCAGAACT
ACE	CTGGAGACCACTCCCATCCTTTCT	GATGTGGCCATCACATTCGTCAGAT
AT1	GAGGTTGAGTGACATGTTCGAAACC	CGTCATCTGTCTAATGCAAAATGTG
ACE2	GTGGTGGGAGATGAAGCGAG	GGGCCTTCATGTTTAGCTGC
GAPDH	GCACCGTCAAGGCTGAGAAC	TGGTGAAGACGCCAGTGGA
miR-6869-5p	GTGAGTAGTGGCGCGCGGC	CTCTACAGCTATATTGCCAG
miR-4466	GGGTGCGGGCCGGCGG	CTCTACAGCTATATTGCCAG
miR-3960	GGCGGCGGCGGAGGCGGGG	CTCTACAGCTATATTGCCAG
miR-122-5p	TGGAGTGTGACAATGGTGT	CTCTACAGCTATATTGCCAG
miR-3663-3p	TGAGCACCACACAGGCCGG	CTCTACAGCTATATTGCCAG
miR-3168	GAGTTCTACAGTCAGAC	CTCTACAGCTATATTGCCAG
miR-4285	GCGGCGAGTCCGACTCAT	CTCTACAGCTATATTGCCAG
U6	CTCGCTTCGGCAGCACAT	TTTGCGTGTCATCCTTGCG

*RT-qPCR, reverse transcription-quantitative polymerase chain reaction; AGT, angiotensinogen; ACE, angiotensin-converting enzyme; AT1, angiotensin 1; ACE2, angiotensin-converting enzyme 2; GAPDH, glyceraldehyde phosphate dehydrogenase; miR, microRNA*.

### Western Blotting

Protein samples from PTECs (miR-6869-5p-transfected and EV-treated cells) were analyzed by Western blotting, as previously described (19, 33, 34). We used the following primary antibodies against several EV-characteristic markers: CD9 (#ab92726, Abcam, Cambridge, MA, USA), CD81 (#sc-7637, 1:200, Santa Cruz Biotechnology, USA), CD63 (#ab59479, Abcam, 1:1,000), AGT (AF3156, R&D Systems, Minneapolis, MN, 1:1,000), ACE2 (AF333, R&D Systems, 1:1,000), ACE (AF929, R&D Systems, 1:1,000), AT1 (MAB102441, R&D Systems, 1:1,000), KIM-1 (NBP1-76701SS, Novus, 1:1,000), and NGAL (AF1757-SP, R&D Systems, 1:1,000). The membranes were subsequently incubated with the corresponding horseradish peroxidase-conjugated secondary antibodies (1:3,000, Cell Signaling Technology). Protein signals were detected by enhanced chemiluminescence (Genshare, China) and quantified using a Bio-Rad imaging system (Hercules, USA). β-actin (#4970, Cell Signaling Technology, 1:3,000) served as the loading control.

### Immunohistochemistry

Animal kidney tissue sections (4 μm) from 4% paraformaldehyde-fixed, paraffin-embedded tissue blocks were prepared for immunohistochemical staining.

The sections were incubated with primary antibodies for KIM-1, NGAL, AGT, and AT1, as indicated above. After washing, sections were incubated with streptavidin–peroxidase-conjugated secondary antibodies (ZSGB-Bio, Inc., Beijing, China). Images were obtained with a DM IL LED microscope (Leica Microsystems GmbH).

### Statistics

Statistical analyses were performed using GraphPad Prism 8.0 (GraphPad Software Inc., San Diego, CA). Data are presented as mean ± standard deviation. Student's unpaired *t*-tests were performed for comparisons between two groups. One-way ANOVA followed by *post hoc* Bonferroni tests were used for multiple comparisons. All experiments were repeated at least three times (all single experiments have a technical duplicate). *P* < 0.05 was considered statistically significant.

## Results

### The Presence of Renal Tubule Damage and RAS Activation in Obesity

To illustrate the presence of renal tubule damage and RAS activation in obesity, baseline data were collected from obese and lean populations (*n* = 25 each group). Table 2 shows that the body mass index, blood glucose, triglycerides, and serum uric acid were increased in obese subjects compared with the lean group (*p* < 0.05). Ambulatory blood pressure, total cholesterol, serum creatinine, cystatin C, and the albumin-to-creatinine ratio (ACR) were similar in both groups. For the systemic RAS, we observed that plasma renin, angiotensin I, and angiotensin II were significantly increased in obese subjects (*p* < 0.05). Next, we established an obese model in C57BL/6J mice. After 18 weeks, the bodyweight of the obese mice was about twice that of the lean mice (Figures 1A–C). In the renal tissue of obese mice, immunostaining was performed to examine the expression levels of renal tubule injury and RAS-related proteins *in vivo*. As shown in Figure 1E, KIM1 and NGAL, which are markers of renal tubular injury, showed enhanced staining in renal tubular epithelial cells in obese mice compared to the lean mice (*p* < 0.05). Our results demonstrate positive immunolabeling for AGT and AT1 in kidneys from obese mice (Figure 1D, *p* < 0.05), confirming the presence of local RAS in obesity. These results were consistent with plasma RAS levels and indicated the occurrence of local RAS activation and renal tubule injury in obese individuals.

**Table 2:** Baseline characteristics of study participants (mean ± SD).

**Characteristic**	**Lean group *N* = 25**	**Obese group *N* = 25**	***p*-Value**
Gender (Male:Female)	11:14	13:12	
Age	28 ± 3	31 ± 10	0.501
BMI	22.53 ± 2.04	40.61 ± 8.40	<0.001
SBP (mmHg)	123 ± 5	128 ± 6	0.116
DBP (mmHg)	70 ± 5	73 ± 6	0.224
Fasting glucose (mmol/L)	4.82 ± 0.75	6.43 ± 2.75	0.023
Triglyceride (mmol/L)	1.00 ± 0.37	2.15 ± 1.90	0.005
Total cholesterol (mmol/L)	4.40 ± 0.47	4.81 ± 1.04	0.160
Serum creatinine (mmol/L)	62.25 ± 15.06	59.87 ± 16.53	0.221
Cystatin C (mg/L)	0.73 ± 0.17	1.07 ± 0.29	0.067
Serum uric acid (mmol/L)	354.1 ± 61.9	459.0 ± 122.8	<0.001
Angiotensin I (ng/ml/h)	0.63 ± 0.22	4.65 ± 3.98	0.007
Angiotensin II (pg/ml)	33.36 ± 7.81	74.83 ± 26.88	<0.001
Renin (ng/ml/h)	0.34 ± 0.24	3.14 ± 2.90	0.024
ACR(mg/g)	14.73 ± 4.10	58.14 ± 38.36	0.154

*Group comparison by Student t-test for independent samples. SBP, systolic blood pressure; DBP, diastolic blood pressure; BMI, body mass index; ACR, albumin-to-creatinine ratio. A P-value of < 0.05 was considered statistically significant*.

### Identification of Plasma EVs From Obese Patients

**Figure 2:**
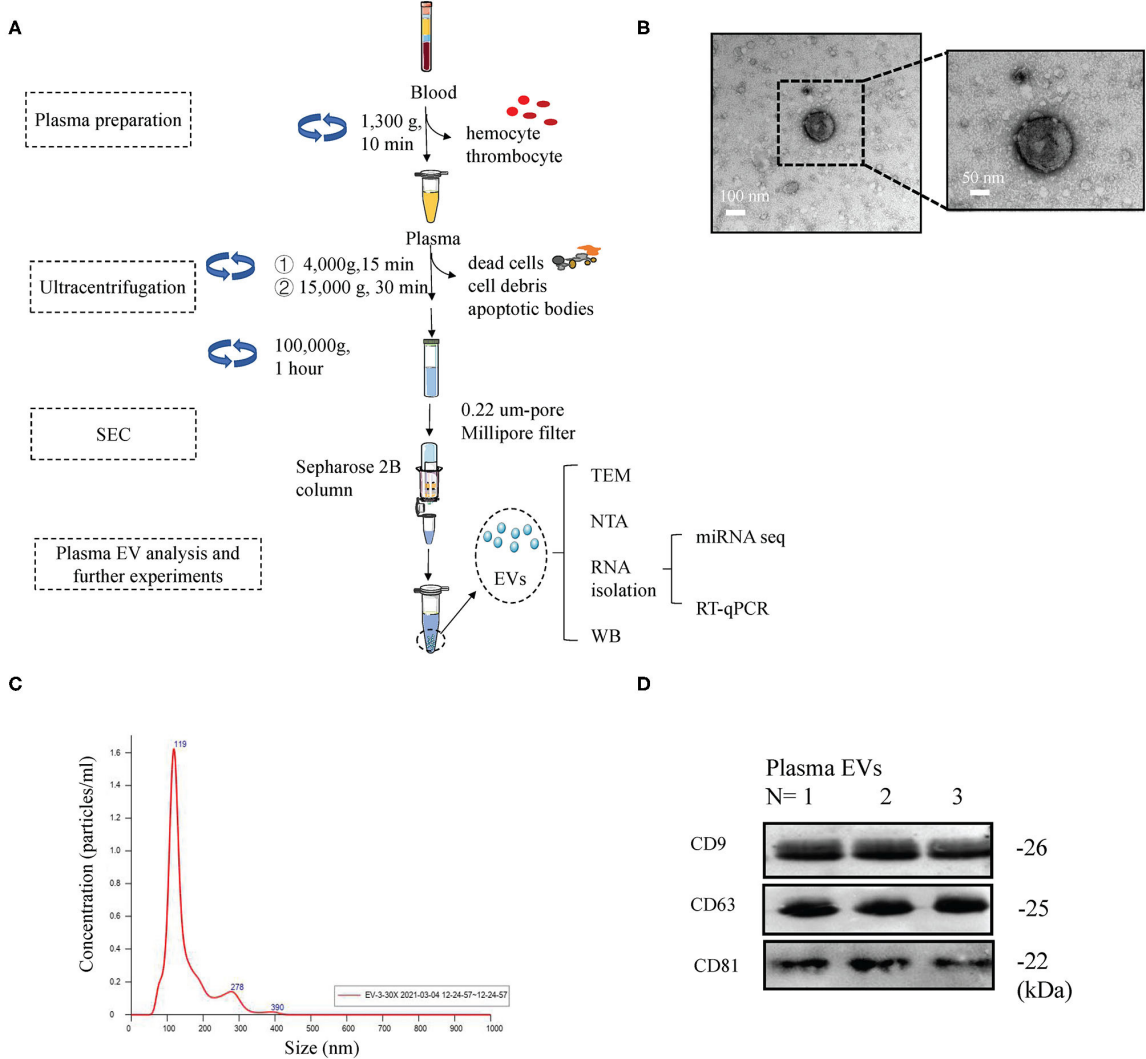
Characterization of plasma EVs in obesity. **(A)** Procedure of EVs isolation from plasma. After collecting plasma samples, EVs were isolated using ultracentrifugation, followed by size-exclusion chromatography. **(B)** Transmission electron microscopy images of plasma EVs from obese patients; Scale bars = 100 nm and 50 nm. **(C)** Nanoparticle tracking analysis showing the concentration and size of plasma EVs from obese patients. **(D)** Western blotting results demonstrated the expression of EVs markers (CD9, CD63, and CD81) in plasma EVs in obese patients. EVs, extracellular vesicles; SEC, size-exclusion chromatography; TEM, transmission electron microscopy; WB, Western blotting; NTA, nanoparticle tracking analysis.

Peripheral blood from five healthy donors and five obese patients was collected following standard procedures to minimize contamination by platelets and platelet-derived vesicles. The remaining plasma was used to isolate EVs. Plasma EVs were isolated using differential ultracentrifugation and modified size-exclusion chromatography (Figure 2A). Transmission electron microscopy images showed that plasma Obese-EVs had cup-shaped or spherical morphology (Figure 2B). Nanoparticle tracking analysis showed that the particle size distribution curve of plasma Obese-EVs was mainly concentrated at 119 nm (Figure 2C). Additionally, EV-associated protein markers, three transmembrane/lipid-bound proteins (CD9, CD63, and CD81) were detected in the particles from obese patient plasma (Figure 2D). Thus, the isolated EVs were putative exosomes, according to standard EV characteristics.

### Effects of Plasma EVs on Renal Tubule Injury and RAS Activation in Obesity

**Figure 3:**
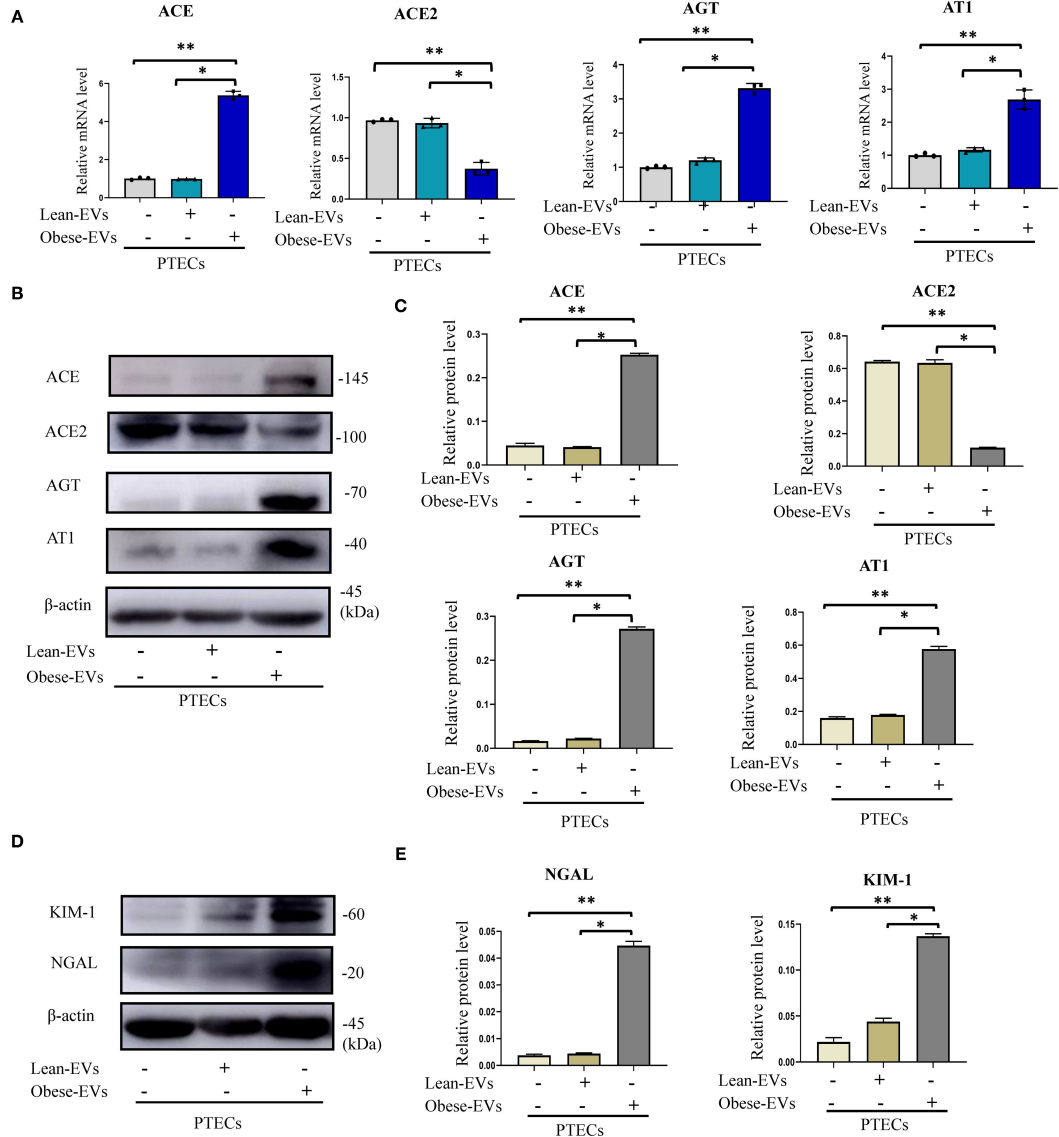
Effects of plasma EVs on renal tubule injury and RAS activation in obesity. **(A–C)** After treating PTECs with PBS(negative control), Lean-EVs, or Obese-EVs, the relative mRNA and protein levels of AGT, ACE, ACE2, and AT1 were analyzed by qRT-PCR **(A)** and Western blotting **(B,C)** (*n* = 3 per group). **(D,E)** Levels of KIM1 and NGAL protein in PTECs treated with PBS, Lean-EVs, or Obese-EVs. Data are presented as mean ± SD; **P < 0.001 vs. PBS, *P < 0.001 vs. Lean-EVs. EVs, extracellular vesicles; PTECs, Proximal tubular epithelial cells; KIM-1, kidney injury molecule-1; NGAL, neutrophil gelatinase-associated lipocalin; AGT, angiotensinogen; ACE, angiotensin-converting enzyme; ACE2, angiotensin-converting enzyme 2; AT1, angiotensin 1.

To investigate the effect of plasma Obese-EVs on RAS activation and renal tubule injury, we analyzed PTECs after incubation with PBS, Lean-EVs (20 μg/mL), or Obese-EVs (20 μg/mL) for 24 h. The Obese-EVs group had 3-fold increased AGT, ACE, and AT1 mRNA expression compared to the Lean-EVs group. In contrast, mRNA for ACE2, a RAS inhibitor, was decreased (Figure 3A, *p* < 0.001). The expression of RAS protein components (ACE, ACE2, AGT, and AT1) was consistent with the mRNA results (Figures 3B,C, *p* < 0.001). Meanwhile, KIM1 and NGAL protein expression were significantly increased in the Obese-EVs group (Figures 3D,E, *p* < 0.001). These findings suggest that Obese-EVs cause renal tubule injury and RAS activation in PTECs.

### Detection of miRNAs in EVs From Obese Patient Plasma

**Figure 4:**
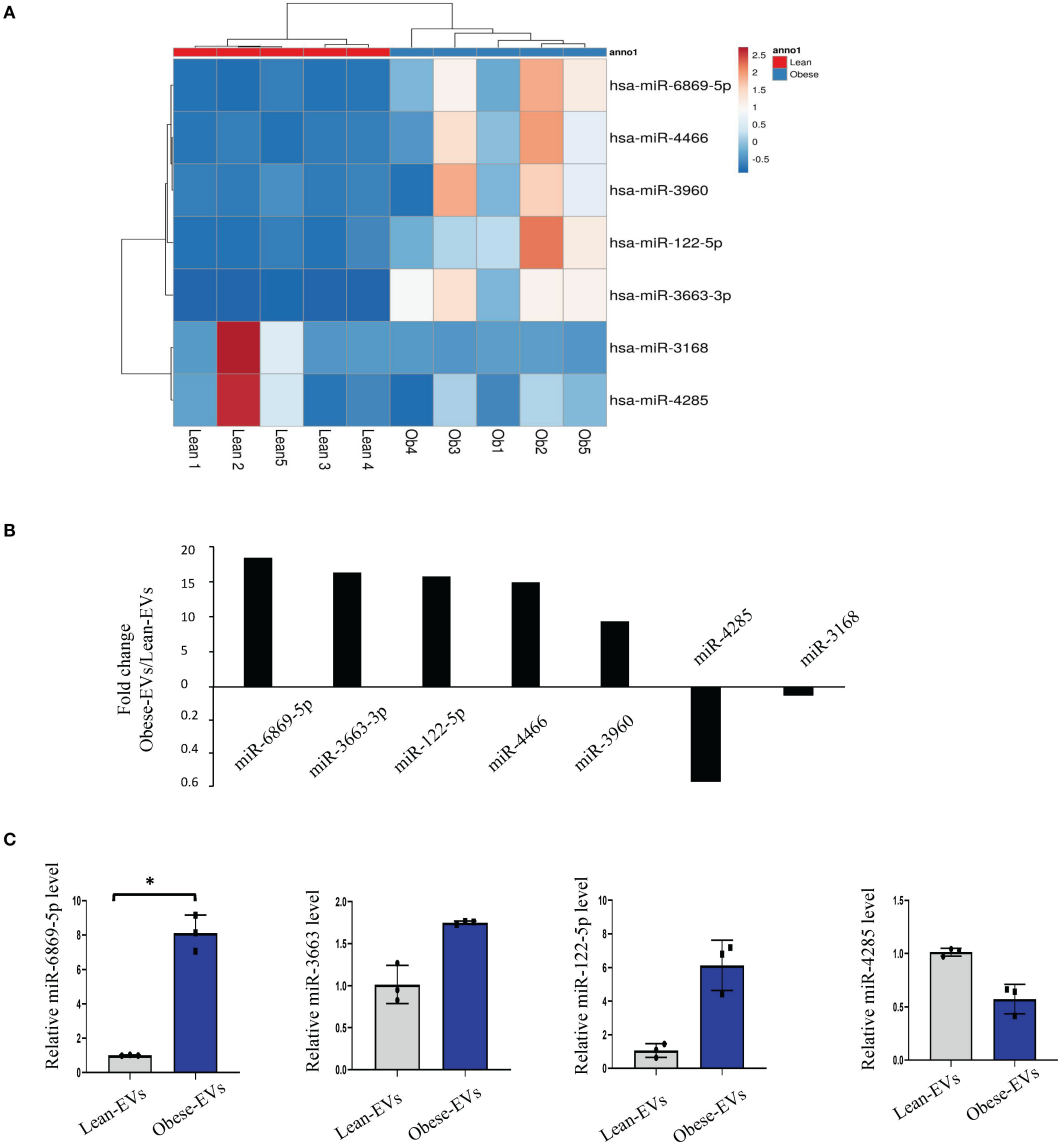
Detection of miRNAs in plasma obese-EVs. **(A)** Heat map of miRNAs. Five miRNAs were upregulated, and two were downregulated in Obese-EVs compared to Lean-EVs. **(B)** The expression of candidate miRNAs was consistent with the miRNA-seq findings. miR-6869-5p, miR-3663, and miR-122-5p were upregulated, while miR-4285 was downregulated in Obese-EVs compared to Lean-EVs. **(C)** Fold-change (Obese-EVs vs. Lean-EVs) of the significantly different miRNAs. (*n* = 3; *P < 0.001 vs. Lean-EVs). EVs, extracellular vesicles.

EVs enable cell-to-cell communication by delivering miRNAs. To explore the key molecules that mediate the disease-causing potential of plasma Obese-EVs in renal tubule injury and RAS activation, we performed microRNA sequencing of plasma EVs from lean and obese patients. We identified several differentially-expressed miRNAs, including hsa-miR-6869-5p, hsa-miR-3663-3p, hsa-miR-122-5p, hsa-miR-4466, hsa-miR-3960, hsa-miR-4286, and hsa-miR-3168 (Figures 4A,B). Moreover, we verified that miR-6869-5p was the most significantly increased in plasma Obese-EVs. miR-6869-5p in Obese-EVs was 5-fold higher than in Lean-EVs (Figure 4C, *p* < 0.001). Therefore, miR-6869-5p probably acts as a strong candidate for the critical regulatory cargo contained in plasma Obese-EVs.

### Obese-EVs Can Deliver miR-6869-5p Into PTECs

**Figure 5:**
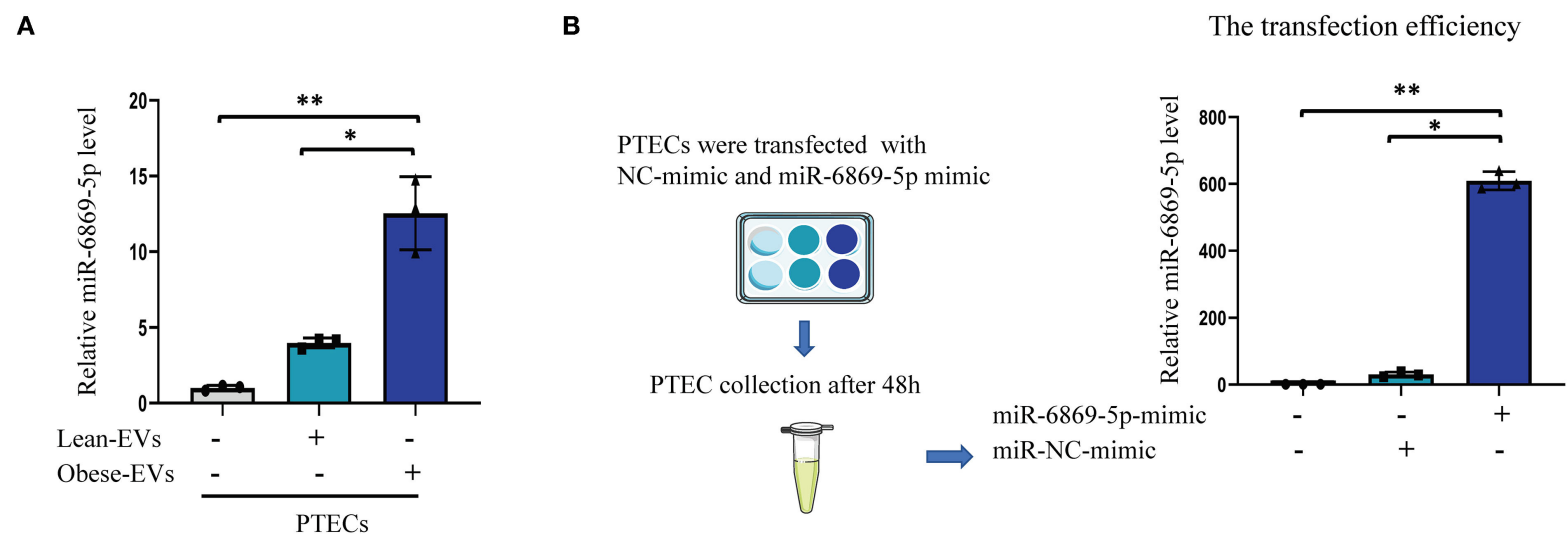
Plasma EVs transport miR-6869-5p into PTECs *in vitro*. **(A)** Quantification of miR6869-5P levels in PTECs after co-culture with PBS, Lean-EVs, or Obese-EVs (*n* = 3). **(B)** The transfection efficiency of the miR-6869-5p mimic was determined by RT-qPCR (*n* = 3). Data are presented as mean ± SD; **P < 0.001 vs. PBS, *P < 0.001 vs. Lean-EVs. EVs, extracellular vesicles; PTECs, Proximal tubular epithelial cells.

To determine whether Obese-EVs can be taken up by PTECs and deliver miR-6869-5p *in vitro*, plasma Obese-EVs and Lean-EVs were incubated with PTECs for 24 h. qPCR analysis revealed that miR-6869-5p levels in PTECs were increased after incubation with Obese-EVs (Figure 5A, *p* < 0.001). These results suggest that miR-6869-5p is shuttled into target cells by Obese-EVs. Next, we transfected a miR-6869-5p mimic into PTECs and measured the transfection efficiency by qPCR. Figure 5B shows that miR-6869-5p in PTECs was 100-fold higher after incubated with miR-6869-5p mimic compared to the NC mimic-transfected group (*p* < 0.001).

### Plasma Obese-EVs Induce Renal Tubule Injury and RAS Activation in PTECs *via* miR-6869-5p Transfer

**Figure 6:**
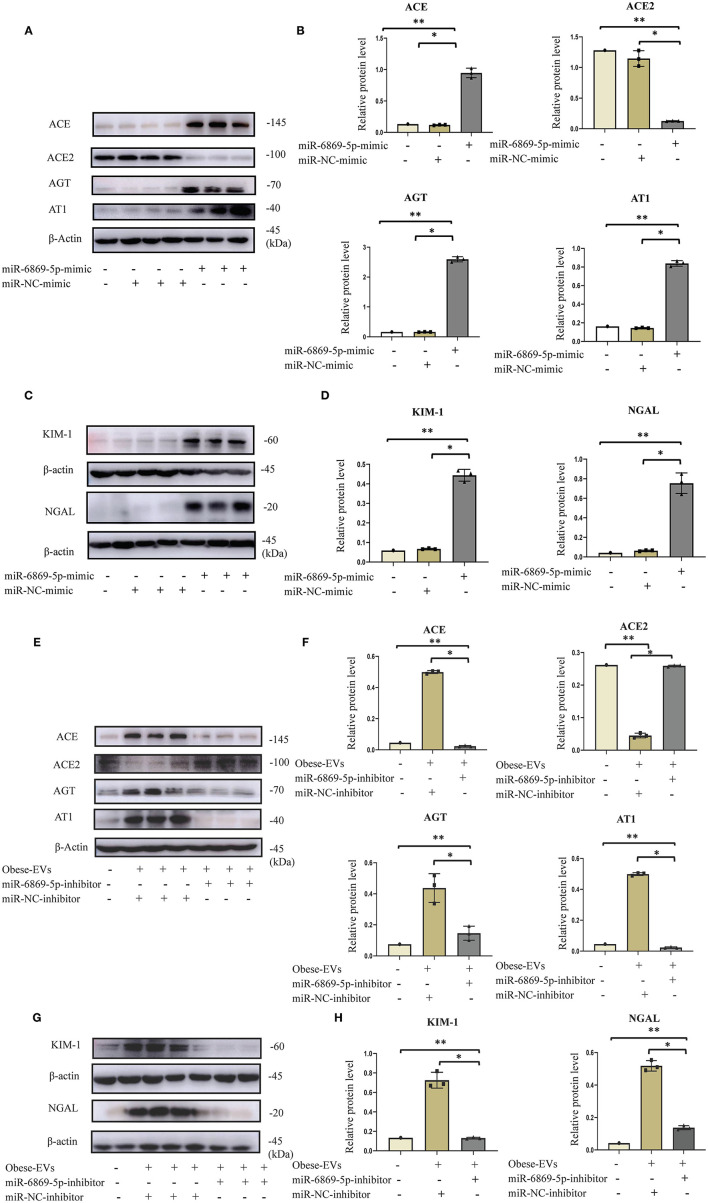
Plasma Obese-EVs induce renal tubule injury and RAS activation in PTECs via transport of miR-6869-5p. **(A–D)** After *in vitro* transfection of miR-6869-5p mimic and miR-NC-mimic, the protein levels of AGT, ACE, ACE2, AT1 **(A,B)**, KIM-I, and NAGAL **(C,D)** were analyzed by Western blotting (*n* = 3). **(E,F)** After transfection with miR-6869-5p inhibitor or miR-NC-inhibitor for 48 h, PTECs were treated with PBS, Lean-EVs, or Obese-EVs. The protein levels of AGT, ACE, ACE2, AT1 **(E,F)**, KIM-I, and NGAL **(G,H)** were analyzed by Western blotting (*n* = 3). Data are presented as mean ± SD; **P <0.001 vs. PBS, *P <0.001 vs. Lean-EVs. EVs, extracellular vesicles; PTECs, Proximal tubular epithelial cells; AGT, angiotensinogen; ACE, angiotensin-converting enzyme; ACE2, angiotensin-converting enzyme 2; AT1, angiotensin 1; KIM-1, kidney injury molecule-1; NGAL, neutrophil gelatinase-associated lipocalin.

We showed that miR6859-5p is enriched in plasma Obese-EVs, and Obese-EVs induce renal tubule injury and RAS activation in PTECs. These two results gave rise to our hypothesis that Obese-EVs mediate miR-6869-5p transfer. MiR-6869-5p might be a critical factor in renal tubule injury and RAS activation. To test this hypothesis, we transfected miR-6869-5p mimic into PTECs. Then, western blot analysis was used to determine the expression of RAS-related components (AGT, ACE, AT1, and ACE2), KIM1, and NGAL. MiR-6869-5p mimic significantly increased the expression of AGT, AT1, ACE, KIM1, and NGAL protein. In contrast, ACE2 expression was significantly reduced (Figures 6A–D, *p* < 0.001). These results were consistent with plasma Obese-EVs activity in PTECs.

**Figure 7:**
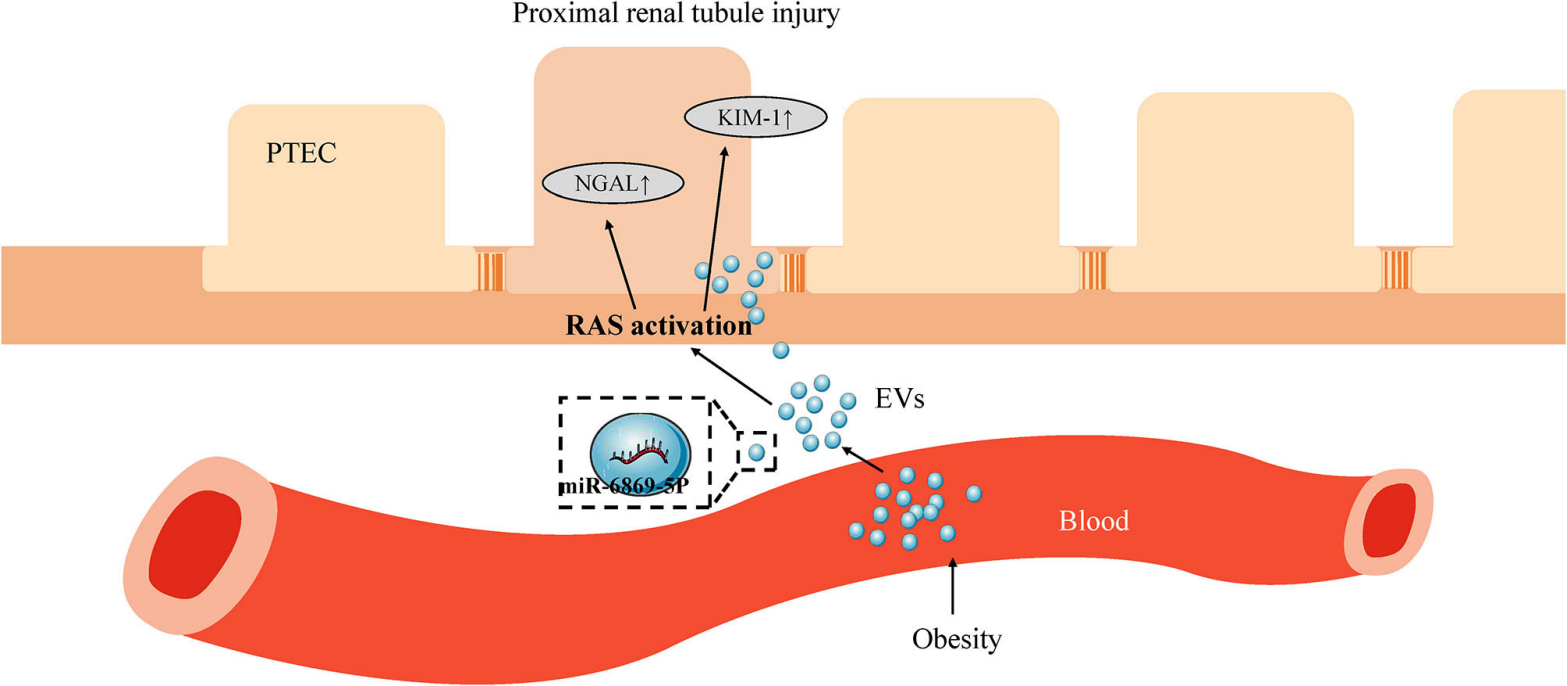
Scheme depicting the role of plasma EV miR-6869-5p and RAS in obesity-related renal tubule injury. In obesity, plasma EVs transport miR-6869-5p into PTECs. miR-6869-5p activates RAS and promotes the expression of KIM-1 and NGAL, markers of renal tubule injury. EVs, extracellular vesicles; PTECs, Proximal tubular epithelial cells; RAS, renin-angiotensin system; KIM-1, kidney injury molecule-1; NGAL, neutrophil gelatinase-associated lipocalin.

To verify the role of miR-6869-5p in Obese-EVs-induced renal tubule injury and RAS activation, Obese-EVs-treated PTECs were treated with a specific inhibitor (miR-6869-5p inhibitor) targeting miR-6869-5p. The results revealed that renal tubule injury and RAS activation induced by plasma Obese-EVs was attenuated by the miR- 6869-5p inhibitor. Western blot analysis demonstrated that AGT, AT1, ACE, KIM1, and NGAL protein levels in PTECs were markedly reduced when treated with miR-6869-5p inhibitor. On the contrary, ACE2 levels were increased (Figures 6E–H, *p* < 0.001). Our results suggest that miR-6869-5p from plasma Obese-EVs mediates renal tubule injury and RAS activation in obesity (Figure 7).

## Discussion

Recent *in vivo* studies demonstrated that EVs play a crucial role in renal diseases. It can contribute to the development of renal diseases by immunomodulation, thrombogenesis, and matrix modulation (6). Renal tubule-derived exosomes play a central role in mediating kidney fibrosis (35). But whether EVs are involved in obesity-related renal injury remains unknown.

Our study showed that plasma Obese-EVs participate in RAS activation and renal tubule damage. Specifically, circulating RAS was activated in obesity. This phenomenon was mainly manifested by increased plasma renin, angiotensin I, and angiotensin II in obese patients. At the same time, local RAS was also activated. We observed increased expression of the RAS-related components AGT and AT1 in the kidney tissue of obese mice. Similarly, the expression of the renal tubular injury markers KIM1 and NGAL also increased. Thus, plasma Obese-EVs mediate RAS activation and renal tubular injury in obesity. Therefore, we confirmed that plasma Obese-EVs are involved in RAS activation and renal tubular damage in obesity. Finally, we show that plasma Obese-EVs carry miR-6869-5p, which causes RAS activation and damage to renal tubular epithelial cells.

Obesity causes various structural, hemodynamic, and metabolic alterations in the kidney. The changes in renal hemodynamics during obesity are closely linked to increased salt sensitivity (36). One important mechanism by which salt sensitivity was increased in obesity was an activation of the intrarenal renin-angiotensin-aldosterone system (RAAS) (37). Our study found obese participants had significantly higher body mass index, blood pressure, blood glucose, total cholesterol, triglycerides, and serum uric acid. Compared with the control group, the serum RAS level had an upward trend. In the kidney tissues of obese mice, the expression of RAS-related components (AGT, AT1) increased. These results are consistent with previous research showing that plasma RAAS concentrations in obese subjects are raised, are correlated with visceral fat mass, and are decreased by weight loss (38, 39). In obese individuals, RAS was activated in renal tissue (40). At the same time, we found that renal tubular damage markers, KIM1 and NGAL, increased along with RAS activation in obese kidney tissue, suggesting that RAS activation might be related to renal tubular damage in obese individuals. Several studies corroborate this result. Inhibition of AT1R and AT2R suppresses renal tubular epithelial cell necroptosis in Ang II-treated renal injury mice (41).

EVs are cell-derived mixed-populations of vesicles released by almost all cells into the intercellular microenvironment. Most of these EVs are located in the blood. Plasma Obese-EVs can induce RAS activation in PTECs. In our experiment, we isolated, quantified, and characterized EVs from plasma in obese and lean participants. Recent evidence suggests a novel role for EVs as conveyors of information among cells and across tissues (14, 42). In our study, after treating PTECs with Obese-EVs, RAS components including AGT, AT1, and ACE, and renal injury biomarkers, KIM1 and NGAL, were significantly increased compared with the Lean-EVs group. Thus, we speculated the plasma EVs could be involved in RAS activation and renal tubular injury during obesity. Significantly, EVs facilitate material transfer and functional communication inside and outside cells (43), partly due to the nucleic acid molecules carried in EVs, especially miRNA (44, 45). Obese-EVs contained microRNAs that target RAS mRNAs and are responsible for RAS activation and renal damage.

Based on these results, miRNA sequencing was performed to identify the miRNAs that play a crucial role in this process. In particular, miR-6869-5p was significantly upregulated in plasma Obese-EVs. Interestingly, the expression of miR-6869-5p robustly increased when PTECs were treated with Obese-EVs. Therefore, we hypothesized that plasma Obese-EVs deliver miR6869-5p into PTECs, but whether miRNA6869-5p plays an important role in renal tubular damage and RAS activation via Obese-EVs transport needs to be further explored.

We demonstrated RAS activation and tubule damage in PTECs by using a miR-6869-5p mimic to stimulate PTECs. These results were consistent with results from treating PTECs with plasma Obese-EVs. Interestingly, the effect of plasma Obese-EVs on RAS activation and renal tubule injury was prevented by a miR-6869-5p inhibitor. Therefore, miR-6869-5p transported by plasma Obese-EVs causes RAS activation and damage to renal tubular epithelial cells. To the best of our knowledge, miR-6869-5p is a newly discovered microRNA involved in regulating diverse cancer cells (46). Accordingly, it was reported that serum exosomal miR-6869-5p might serve as a tumor suppressor in colorectal cancer. The protective effect of miR-6869-5p against colorectal carcinogenesis is dependent on the TLR4/NF-κB signaling pathway (47). At the same time, Wang et al. found that miR-6869-5p plays beneficial roles in inhibiting glioma cell proliferation and invasion (48). As yet, the role of EVs and miR-6869-5p in non-cancerous diseases remained unclear. To address this gap, we explored the role of miR-6869-5p in obesity and provided the first evidence that plasma Obese-EVs act as a paracrine mediator in obesity-related kidney disease.

Our study also has some limitations. First, we need to identify relevant mRNA targets of miR-6869-5p and clarify the specific mechanism between miR-6869-5p and RAS activation. Moreover, miR-6869-5p is the novel microRNA that was first identified in humans. A future study will address differential miR-6869-5p expression in animal models and verify our results *in vivo*. We will further search for the targeting pathways of miR-6869-5p in renal tubule RAS activation through the next experiments.

In conclusion, we demonstrate that plasma Obese-EVs induce renal tubule injury and RAS activation via miR-6869-5p transport (Figure 7). Thus, miR-6869-5p within plasma Obese-EVs could be a therapeutic target for local RAAS activation in obesity-associated kidney disease.

## Data Availability Statement

The datasets presented in this study can be found in online repositories. The names of the repository/repositories and accession number(s) can be found below: GenBank database. The submission number is SUB9882595. The BioProject number is PRJNA739404.

## Ethics Statement

The studies involving human participants were reviewed and approved by the Ethics Committee of the First Affiliated Hospital of Jinan University (Permission No. 2018-041). The patients/participants provided their written informed consent to participate in this study. The animal study was reviewed and approved by the Ethics Committee of the First Affiliated Hospital of Jinan University (Permission No. 2018-041).

## Author Contributions

YM and C-CW designed the studies and supervised the project. H-HL, X-QL, and J-FL performed most of the experiments and co-wrote the manuscript. S-BH and WY assisted with the clinical data collection. HL performed the EV isolation and identification. SC performed the histological analysis. LW performed the bioinformatics analysis. BH analyzed the data and provided statistical guidance. All authors contributed to the article and approved the submitted version.

## Funding

This research was funded by the Natural Science Foundation of Guangdong, China (2018A030313527); The Basic and Applied basic research Foundation of Guangdong Province, China (2019A1515010176); The Science and Technology Project of Guangzhou, China (202102010133); The Science and Technology Project of Shenzhen, China (JCYJ20190808095615389); and the National Natural Science Foundation of China (82000686).

## Conflict of Interest

The authors declare that the research was conducted in the absence of any commercial or financial relationships that could be construed as a potential conflict of interest.

## Publisher's Note

All claims expressed in this article are solely those of the authors and do not necessarily represent those of their affiliated organizations, or those of the publisher, the editors and the reviewers. Any product that may be evaluated in this article, or claim that may be made by its manufacturer, is not guaranteed or endorsed by the publisher.
